# Estimating proportion of days covered (PDC) using real-world online medicine suppliers’ datasets

**DOI:** 10.1186/s40545-021-00385-w

**Published:** 2021-12-29

**Authors:** David Prieto-Merino, Amy Mulick, Craig Armstrong, Helen Hoult, Scott Fawcett, Lina Eliasson, Sarah Clifford

**Affiliations:** 1 Sprout Health Solutions Ltd, London, UK; 2 London School of Hygiene and Tropical Medicine, London, UK; 3 Pharmacy2U, Leeds, UK

**Keywords:** Medication adherence, Proportion of days covered, Real-world data, Measurement, Routinely collected data

## Abstract

**Background:**

The proportion of days covered (PDC) is used to estimate medication adherence by looking at the proportion of days in which a person has access to the medication, over a given period of interest. This study aimed to adapt the PDC algorithm to allow for plausible assumptions about prescription refill behaviour when applied to data from online pharmacy suppliers.

**Methods:**

Three PDC algorithms, the conventional approach (PDC1) and two alternative approaches (PDC2 and PDC3), were used to estimate adherence in a real-world dataset from an online pharmacy. Each algorithm has different denominators and increasing levels of complexity. PDC1, the conventional approach, is the total number of days between first dispensation and a defined end date. PDC2 counts the days until the end of supply date. PDC3 removes from the denominator specifically defined large gaps between refills, which could indicate legitimate reasons for treatment discontinuation. The distribution of the three PDCs across four different follow-up lengths was compared.

**Results:**

The dataset included people taking ACE inhibitors (*n* = 65,905), statins (*n* = 100,362), and/or thyroid hormones (*n* = 30,637). The proportion of people taking ACE inhibitors with PDC ≥ 0.8 was 50–74% for PDC1, 81–91% for PDC2, and 86–100% for PDC3 with values depending on drug and length of follow-up. Similar ranges were identified in people taking statins and thyroid hormones.

**Conclusion:**

These algorithms enable researchers and healthcare providers to assess pharmacy services and individual levels of adherence in real-world databases, particularly in settings where people may switch between different suppliers of medicines, meaning an individual supplier’s data may show temporary but legitimate gaps in access to medication. Accurately identifying problems with adherence provides the foundation for opportunities to improve experience, adherence and outcomes and to reduce medicines wastage. Research with people taking medications and prescribers is required to validate the algorithms’ assumptions.

**Supplementary Information:**

The online version contains supplementary material available at 10.1186/s40545-021-00385-w.

## Background

The World Health Organization (WHO) has defined adherence as “the extent to which a person’s behaviour—taking medication, following a diet, and/or executing lifestyle changes—corresponds with agreed recommendations from a health care provider” [1]. Poor adherence to treatment can adversely affect people’s health outcomes, present a significant barrier to optimal care and can increase healthcare costs [2, 3]. Previous research suggests that annual waste from unused medications is approximately £300 million per year in England [4], and that improving adherence can help reduce this cost [5]. To tackle the enduring problem of nonadherence to medication, it is important to measure and understand medication adherence accurately.

Over the last decade, there have been improvements in the way that adherence is conceptualised and defined. For example, adherence is now understood as involving several distinct, quantifiable behaviours: initiation is when the person takes the first dose of prescribed medication (this may be the same or different to the prescription date); implementation is the degree to which the person’s intake of medication corresponds to the prescription, from initiation until the last dose taken; discontinuation is the term given to the time at which the person has taken their last dose and no further doses are taken and persistence is the duration of time between the first and last dose taken (from implementation to discontinuation) [6]. This relatively new taxonomy of adherence (the ABC taxonomy) can help researchers and healthcare professionals to better understand, interpret and support medicine-related behaviour. It also provides a rationale for refining measures of adherence, for example by specifying which type of adherence behaviour (e.g. implementation or persistence) is being measured.

Assessing people’s adherence to medication in real-world settings can identify areas of unmet need and potential opportunities to intervene to improve adherence. Adherence assessment and interventions have been well-studied in the areas of traditional primary care and community pharmacy [7, 8], but less so in emerging areas where people seek healthcare and medication, such as online pharmacy providers. The global market for online pharmacies is estimated to increase from around $42.3 billion in 2018 to $107.5 billion by 2025 [9]. Therefore, analysis of electronic pharmacy record data is a promising option for applying proxy adherence measures and for tracking people’s medication use over time [10]. However, to use these data, we need to review and potentially adapt the measures used to assess adherence in more traditional, controlled settings, and to accommodate justifiable variations in the conceptualisation of adherence [6].

Despite a substantial number of potential methods for measuring medication adherence, including self-report, electronic monitoring, and algorithms for pharmacy database analysis, there is no gold standard method [11, 12]. Two methods for estimating adherence from pharmacy databases are commonly used [13–15]. These are the medication possession ratio (MPR) and the proportion of days covered (PDC). While neither measure can confirm that the prescribed medication was ingested or taken as prescribed, both can provide insight into whether the medication was available for the person to take.

The PDC metric has been advocated by the Pharmacy Quality Alliance (PQA) as the preferred quality indicator for estimating adherence to therapies for chronic diseases [16, 17]. The PDC looks at the proportion of days in which the person has access to the medication, over the POI and is generally calculated as: (sum of days covered in the POI) ÷ (number of days in the POI) × 100 [15]. Typically, the threshold for adequate adherence has been placed at 80% for most medications, and 90% for antiretrovirals [1, 18].

There are some problems with the PDC. For example, on the surface, PDC may appear to be a simple calculation. However, the numerator and/or the denominator may incorporate a range of variable definitions depending on the healthcare system’s procedures and payor data requirements. There are important differences between data from the comprehensive electronic systems used in mature e-health markets like Sweden and Denmark, which contain both prescription and dispensing information, and the complex realities of everyday healthcare in systems such as the United Kingdom (UK)’s National Health Service (NHS) [19]. The NHS is a relatively late adopter of electronic prescription services, and the use of any prescription service depends on provider access, patient choice and drug classification [19], resulting in more fragmented data. Over time, people may obtain their prescriptions from different prescribers and redeem their prescriptions from different pharmacies within different networks that do not share records. The introduction of online pharmacies has increased people’s choice of medication suppliers and provides more opportunities for people to obtain their medicines from more than one supplier. There is a gap in the literature on how to apply PDC algorithms in a way that is appropriate for quality assessment in these types of real-world settings.

While many authors have identified potential problems with the PDC [11, 15], few have proposed and tested methods for overcoming them [20]. One potential limitation in the summary measurement provided by the PDC is that it fails to account for different explanations for substantial gaps in treatment [20]. For example, according to the PDC, a substantial gap between two refills is always assumed to be the result of sub-optimal implementation (a treatment holiday), yet there are alternative, feasible explanations, such as a break in prescribing of the medication, or the person could be receiving treatment from another pharmacy. While it is not always possible to determine the reason for a break (and from a patient care perspective, breaks should always trigger an attempt to connect with the patient), methods for accounting for various scenarios are needed.

García-Sempere et al. have explored this problem, using prescribing and dispensing data on osteoporosis treatment that was captured within one large medical system in Spain [20] where data from prescription and purchasing in pharmacies could be linked. However, in many circumstances, linked prescribing and dispensing data are not available. The need remains for a model that has been tested in more than one therapy area, relies on dispensing data only and can account for people who fill their prescriptions (or not) across different organisations.

The aim of this study was to improve the measurement of adherence according to the PDC by introducing and evaluating two novel approaches to defining the denominator for the calculation of the PDC, under varied assumptions, when data are available from just one pharmacy provider. The proposed algorithms are presented and explained using hypothetical examples, followed by application and evaluation in real-world data examples.

## Methods

Institutional review board (IRB) approval was not required for this study because it involved secondary use of unidentifiable information from a commercial provider. Data have been stored electronically and can be requested under a confidentiality agreement.

### Principles of PDC calculation

PDC is calculated within a defined POI. This period can start relative to patient events (e.g. treatment initiation) or calendar dates (e.g. January 1st 2020) and usually ends after a pre-defined length of time (e.g. 1 year). The numerator in all fractions is necessarily the number of days covered by the pharmacy-supplied medication in the POI. This is calculated by summing the total daily doses dispensed during the POI, where the daily dose is the number of medication units dispensed divided by the number of units to be taken per day.

### Denominator

The denominator is the number of days a medication is needed during a POI. Since the pharmacy dispensing the medication cannot know for sure on how many days the medication is ‘needed’ from the data that are available from their system, certain assumptions must be made to calculate the denominator. For each individual, the denominator depends on one or more of the following three variables:

Variable 1: date on which the individual began receiving medication from this provider within the POI.
Variable 2: date on which the individual stopped receiving medication from the pharmacy within the POI. This could be due to, e.g. discontinuation of the medication by their prescriber or by the individual themselves permanently switching services to a competing pharmacy, relocation out of service area, death, and many other reasons. These are assumptions that there is a genuine reason for stopping that is not due to nonadherence.
Variable 3: the magnitude of any temporary breaks in use of this pharmacy by the individual within the POI. Possible explanations for these breaks fall into one of three categories:
  - There is a medically prescribed temporary interruption of treatment.
  - Medicines are temporarily dispensed by another pharmacy.
  - People are not implementing their treatment regime as prescribed (as defined by ABC Taxonomy [6]).

The first two explanations assume genuine reasons for stopping that do not indicate nonadherence.

When using a single supplier’s dataset to study a POI, it is possible to discern the dates when each person first received a medicine (variable 1 above) and when they stopped altogether (variable 2 above). However, it is not possible, in general, to know the reasons why each person stopped ordering from this pharmacy. While the pharmacy can detect temporary breaks in people’s orders (variable 3), it is not usually possible to distinguish whether these breaks are due to a prescribed treatment interruption (variable 3, explanation a), to the use of another pharmacy (variable 3, explanation b) or to nonadherence (variable 3, explanation c). This implies that the PDC calculated with only pharmacy dispensing data may not reflect accurate measures of people’s true adherence. However, it may be possible to estimate different versions of the PDC by making different assumptions about variables 2 and 3 above. If correctly interpreted, these different versions of the PDC may provide additional information about medication adherence, as well as an indication of the provider’s efficiency in supplying medicines, and each version may be best suited to different circumstances.

### PDC1

The usual PDC denominator, which for the purposes of this paper we are calling ‘PDC1’, is the number of days between an individual’s first supply date within the POI and the last day of the POI. This method assumes that individuals are (1) alive and (2) in need of medication from the first supply date to the end of the POI. It assumes the prescription does not change and no other events happen that legitimately exempt people from medication need during the POI. If a person stops refilling their prescription during the POI (variable 2 above), then PDC1 simply ignores that fact.

PDC1 assumes that long breaks between refills or failing to refill prescriptions at this provider indicates people have sub-optimal treatment implementation (variable 3, explanation c). It does not allow for the possibility that there is a medically prescribed temporary interruption of treatment (variable 3, explanation a) or for the possibility that medicines are temporarily dispensed by another pharmacy (variable 3, explanation b).

### Proposal for two extensions to PDC

PDC2 and PDC3 are variations of PDC1, which modify the assumptions when there is insufficient information about the status of people when they are not being observed.

### PDC2

The first proposed adaptation of the traditional PDC algorithm, PDC2, changes the denominator by making different assumptions about individuals’ circumstances. PDC2’s denominator is the number of days between the date each individual obtained their first supply and the date when the last supply obtained runs out within the POI. In contrast to PDC1, PDC2 does not assume individuals are still alive and in need of treatment beyond the period covered by their last refill at this provider. Note that, for individuals who continue their medication until the end of the POI, the PDC2 denominator equals the PDC1 denominator. For individuals who discontinue their medication, the PDC2 denominator is smaller than the PDC1 denominator, thus producing a larger PDC.

PDC2 assumes that long breaks between refills at this pharmacy indicate people have sub-optimal implementation (variable 3, explanation c).

### PDC3

Our second proposed adaptation of PDC, PDC3, extends PDC2 by discounting large gaps between refills. The denominator is the number of days between each individual’s first supply date and the date the last supply would run out within the POI, minus the total number of days contained in ‘large’ gaps between refills.

PDC3 assumes that large gaps between refills at a pharmacy indicate either that individuals are having a medically justified treatment break (variable 3, explanation a) or that they are seeking treatment from other providers and are implementing their treatment as prescribed (variable 3, explanation b).

The difference between PDC3 and the other two PDCs is the assumption that large gaps between refills from a provider do not necessarily indicate nonadherence. In contrast, all three methods, PDC1, PDC2 and PDC3, assume that short gaps between refills do indicate sub-optimal implementation of prescribed treatment, and not that supply is sought elsewhere.

### The definition of large gaps

It is proposed that within variable 3 above, ‘large’ should be defined to indicate an individual is temporarily discontinuing refills from the provider and is not merely late to refill. The definition of a large gap may need to vary according to context, for example the specific drug’s pharmacodynamic and pharmacokinetic profiles and the nature of the disease. As a general example, a gap could be defined as large if the number of ‘uncovered days’ between two orders exceeds the number of ‘covered days’ in the next order.

For example, if the next order is for 28 days’ supply, a large gap would be 28 days or more and the multiplying factor for the gap should be 1. For medications that are not critical for the patient, longer gaps might be allowed before assuming that the patient is obtaining their medicines elsewhere—for example, a factor of 1.5 times the number of days covered by the next prescription. However, if, for example, patients have a life-threatening disease and could die after 3 weeks without the drug, a gap of more than 2 weeks is unlikely to be due to nonadherence, therefore the multiplying factor for the gap should be smaller (for example a factor of 0.5).

Note that if there are no ‘large’ gaps between refills, PDC3 is equivalent to PDC2, which is in turn equivalent to PDC1 if individuals do not discontinue their medication.

**Fig. 1:**
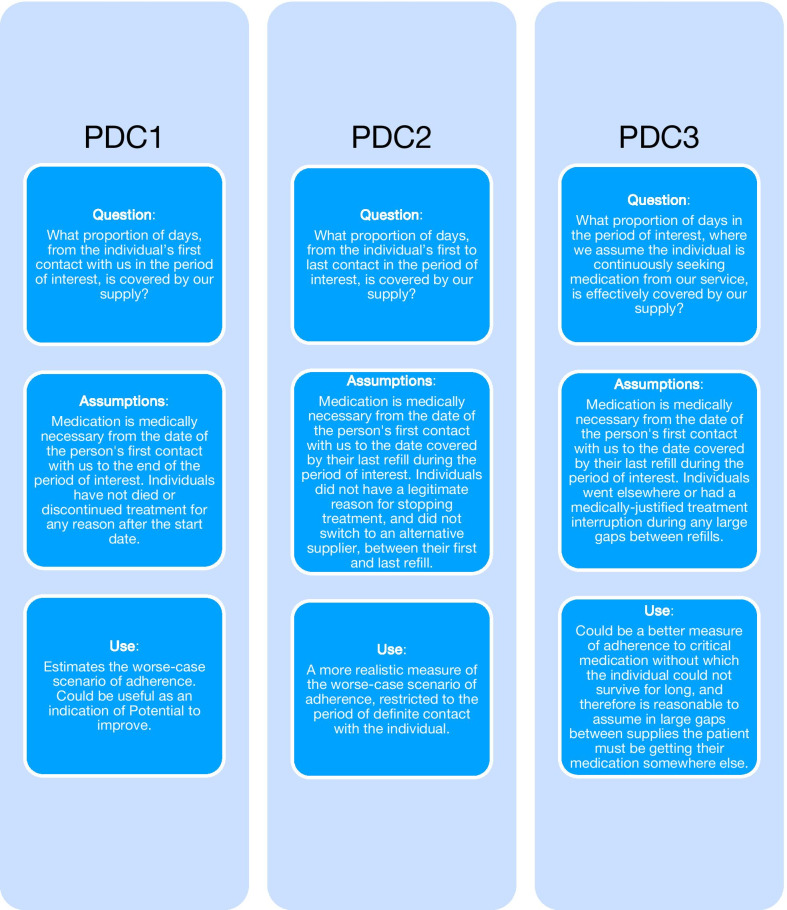
Comparison of research questions and assumptions between the three proposed PDC measures

Figure 1 is a schematic of the three PDC measures and the relevant research questions and assumptions that are made when using them.

### Illustration of the three PDC methods

**Fig. 2:**
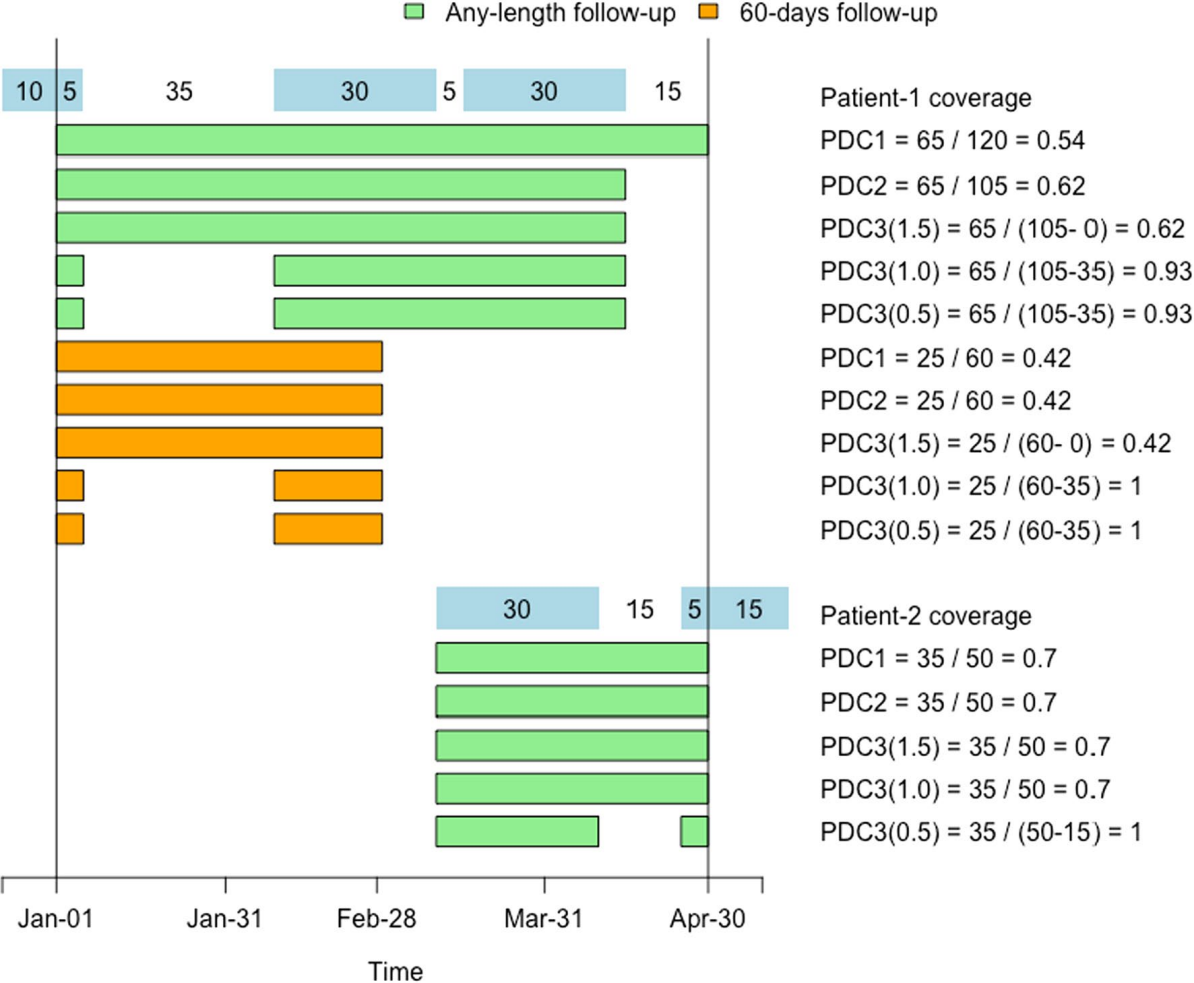
Graphical representation of two patterns of prescription medication supply and calculation of PDC under different follow-up lengths. Period of interest is defined between January 1st and April 30th

Figure 2 highlights differences between the three methods, using an artificial example of two individuals with different patterns of online maintenance prescription usage between January 1st and April 30th of a certain year. PDCs are compared in two POIs: the whole 4-month period and a 60-day sub-period. To compare the PDCs in the 60-day sub-period, the POI is redefined to end at the earliest of 60 days after first supply date or April 30th. An illustration of what happens with PDC3 when the legitimate gap multiplier takes different values is also presented.

The scenario is illustrated for Patient 1. Their first supply date is the first day of the study period because they are already taking medication. For the 4-month period, the PDC1 denominator ends at the end of the POI (day 120) so PDC1 is 65/120 = 0.54. The PDC2 denominator ends earlier at the last day of supply (day 105): PDC2 = 65/105 = 0.62. For PDC3, the multiplier to estimate “large gaps” appears between the round brackets. Using a multiplier of 1.5 the first gap is not considered large, because the number of days in it is less than 1.5 times the number of days’ supply after the gap (35 < 1.5 * 30). Thus PDC3 (1.5) includes the gap in the denominator and produces the same coverage as PDC2. However, PDC3 (1.0) and PDC3 (0.5) consider the gap “too large” (35 > 1 * 30 and 35 > 0.5 * 30, respectively) and exclude it from the denominator calculating a coverage of 65/(105–35) = 0.93 each. Restricting the POI to 60 days, we stop observing this patient on March 1st with 25 days covered. PDC1, PDC2 and PDC3 (1.5) are identical and smaller than with full follow-up because the 35-day gap is now a large proportion of the first 60 days. PDC3 (1.0) and PDC3 (0.5) remove the gap and become 1.

Note that in this example, and generally, PDC1 ≤ PDC2 ≤ PDC3 (1.5) ≤ PDC3 (1.0) ≤ PDC3 (0.5). Therefore, choosing which measure to report depends entirely on the research question, the follow-up period of interest and the assumptions one is willing to make about legitimate gaps between dispatches.

### Example using real-world data

These three PDC algorithms were applied to a real-world dataset from Pharmacy2U, an online pharmacy in the United Kingdom (UK). A subset of their database was selected by Pharmacy2U analysts, according to the following criteria:

- Prescriptions with ‘dispatch date’ within the POI: 1st January 2018 to 31st December 2019.
- Prescriptions containing medications within three drug classes identified for real-world testing: angiotensin-converting enzyme (ACE) inhibitors, statins, or thyroid hormones. These medicines were selected because they were all maintenance medications that would need to be refilled (rather than taken as needed).
- Prescriptions with continuation of agreed dosage instructions of ‘one per day’ or equivalent throughout the study period.
- Only National Health Service (NHS) prescriptions.

The dataset was fully deidentified before it was shared with the study team for analysis. No patient data were shared in a way in which individual patients could be identified. Pharmacy2U’s Privacy Policy allows the use of customer information in pursuit of their legitimate interests of operating and developing their commercial pharmacy services [21].

In each of the three drug categories, each PDC was calculated over four follow-up lengths after the first dispatch date within the POI: 3-month, 6-month, 12-month, and follow-up until the end of the POI (referred to as “Any-month” follow-up). To have a valid PDC calculation for an *X*-month follow-up, a person must have had their first dispatch at least *X* months before the end of the POI. For example, a person who has their first dispatch 5 months before the end of the study period cannot have a PDC calculation for a 12-month or a 6-month follow-up (but they can have PDC for a 3-month follow-up, and for end of study (“any month”) follow-up. PDC3 was calculated using 0.5 (liberal), 1 (moderate) and 1.5 (conservative) times the number of days covered in the prescription immediately after the gap.

## Results

The resulting P2U database consisted of 65,905 people in the UK with prescriptions for ACE inhibitors, 100,362 for statins and 30,637 for thyroid hormones with prescriptions dispatched in the target timeframe. In people taking ACE inhibitors, statins, and thyroid hormones, approximately 40%, 40% and 83% were female and 56%, 69% and 37% were aged 60 or older, respectively (Table 1).

**Table 1:** Sample characteristics

Characteristic	ACE inhibitors	Statins	Thyroid hormones
Gender			
Female	26,229 (39.8%)	39,590 (39.4%)	25,454 (83.1%)
Male	39,676 (60.2%)	60,772 (60.6%)	5183 (16.9%)
Age group			
< 18	75 (0.1%)	39 (0.0%)	216 (0.7%)
18–29	477 (0.7%)	307 (0.3%)	1464 (4.8%)
30–39	2202 (3.3%)	1683 (1.7%)	3939 (12.9%)
40–49	8396 (12.8%)	8102 (8.1%)	6086 (19.9%)
50–59	17,916 (27.2%)	21,407 (21.4%)	7492 (24.5%)
60–69	18,069 (27.5%)	31,995 (31.9%)	5571 (18.2%)
70–79	11,643 (17.7%)	24,153 (24.1%)	3411 (11.1%)
80+	7017 (10.7%)	12,509 (12.5%)	2415 (7.9%)
Region			
East Anglia	8292 (12.6%)	12,527 (12.5%)	5181 (16.9%)
East Midlands	5374 (8.2%)	7815 (7.8%)	2874 (9.4%)
London	6512 (9.9%)	11,665 (11.6%)	3012 (9.8%)
North East	2835 (4.3%)	4260 (4.3%)	1301 (4.3%)
North West	7222 (11.0%)	11,085 (11.1%)	2353 (7.7%)
Northern Ireland	1 (0.0%)	1 (0.0%)	0 (0.0%)
Scotland	7 (0.0%)	8 (0.0%)	2 (0.0%)
South East	15,021 (22.8%)	22,265 (22.2%)	6298 (20.6%)
South West	9192 (14.0%)	12,989 (13.0%)	4451 (14.6%)
Wales	39 (0.1%)	56 (0.1%)	13 (0.0%)
West Midlands	5870 (8.9%)	8908 (8.9%)	2256 (7.4%)
Yorkshire and the Humber	5424 (8.2%)	8606 (8.6%)	2850 (9.3%)
Ordering platform			
App	13,810 (21.0%)	18,453 (18.4%)	6317 (20.6%)
Telephone	4195 (6.4%)	7449 (7.4%)	1450 (4.7%)
IVR	6451 (9.8%)	10,626 (10.6%)	3202 (10.5%)
Direct to surgery	17,134 (26.0%)	27,309 (27.2%)	7922 (25.9%)
WEB	24,318 (36.9%)	36,530 (36.4%)	11,746 (38.3%)
CCG restricted^a^			
No	46,556 (70.6%)	70,805 (70.5%)	21,141 (69.0%)
Yes	19,352 (29.4%)	29,562 (29.5%)	9496 (31.0%)
Registration method			
App	5522 (8.4%)	7374 (7.3%)	2529 (8.3%)
Online	43,808 (66.5%)	63,310 (63.1%)	21,127 (69.0%)
Partnerships	528 (0.8%)	971 (1.0%)	122 (0.4%)
RegForm	11,599 (17.6%)	20,575 (20.5%)	5207 (17.0%)
Telephone	4451 (6.8%)	8137 (8.1%)	1652 (4%)

*IVR* Interactive voice response, *CCG* Clinical Commissioning Group

^a^CCG Restricted is the requirement of certain prescriptions to be requested directly with General Practitioner surgeries rather than via the pharmacy

**Fig. 3:**
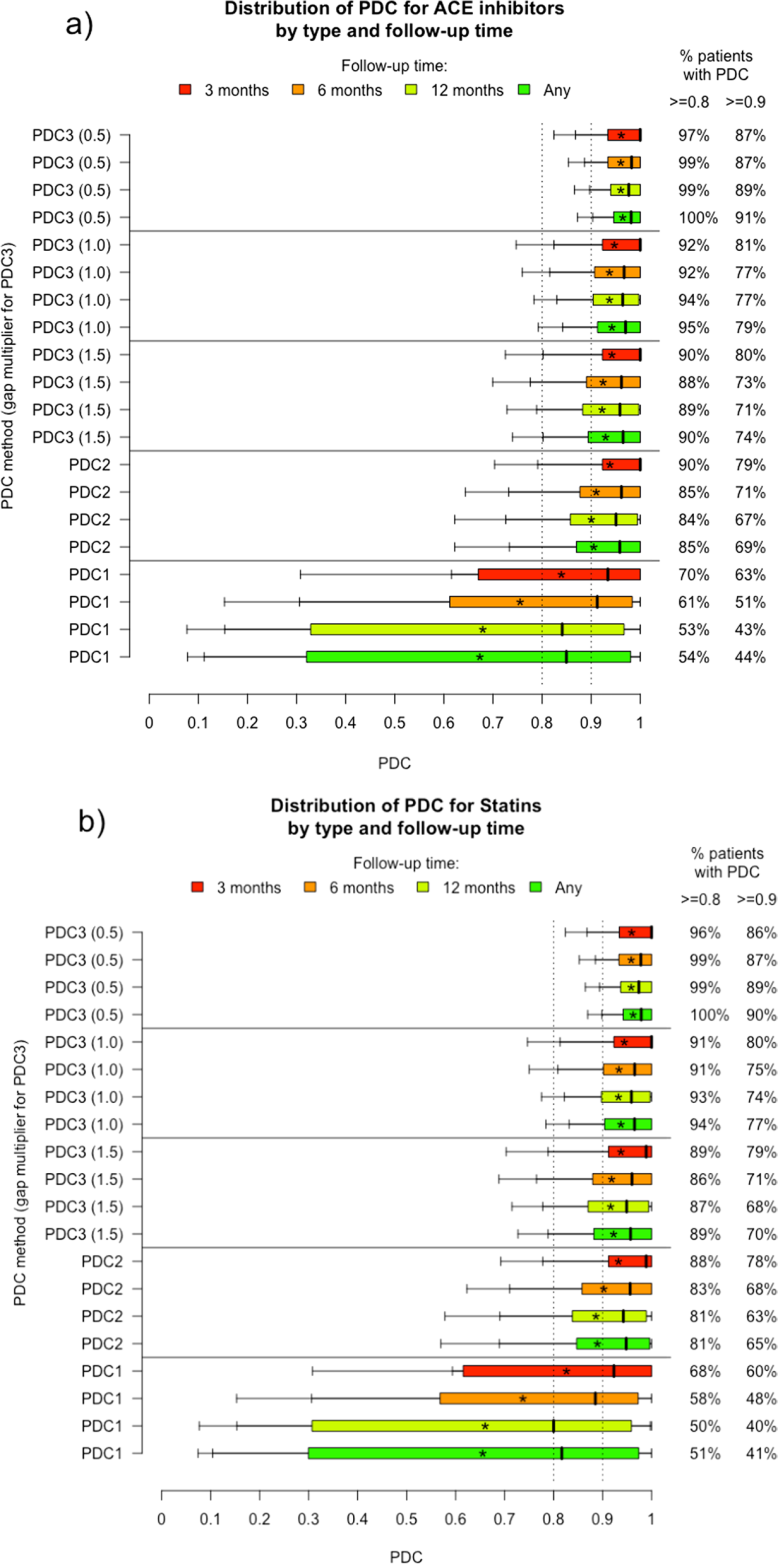

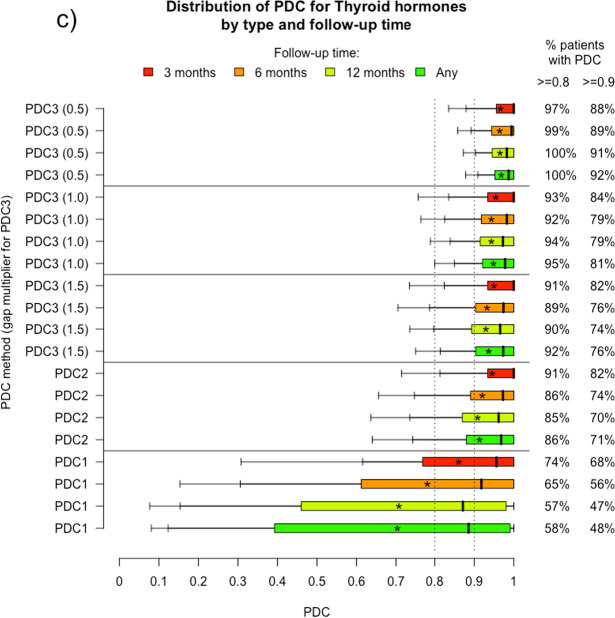
Three PDC algorithms applied to a real-world dataset. PDCs are shown for people taking **a** ACE inhibitors, **b** statins and **c** thyroid hormones. Each boxplot is for a combination of PDC definition (labelled on the left) and follow-up period (coloured and identified in legend). Each shows the 25th and 75th percentiles at either end of the box, the 50th percentile as a black line in the middle, and up to four ‘whiskers’ at the 5th, 10th, 90th and 95th percentiles. The asterisk in each box is the mean. PDC3 is calculated such that gaps of 0.5, 1 and 1.5 relative to the average number of days in each prescription are factored out of the denominator on the assumption that the person has gone elsewhere for their supply during that time

Figure 3 shows boxplots of the three PDCs across four POIs and across three definitions of long gaps for PDC3. In all three drug classes, there are substantially more individuals with a low PDC1 than there are with low PDC2 or PDC3. For “Any” length of follow-up in the study period, in those taking ACE inhibitors the median PDC1 was 0.85 compared to 0.96 for PDC2 and 0.97–0.98 for PDC3 (medians shown as thick vertical black lines in the boxes in Fig. 3 and as data in Additional file 2: Table S1). Similarly, the medians for PDC1–PDC3 in Statins were 0.82, 0.95 and 0.96–0.98, and in thyroid hormones were 0.89, 0.97 and 0.97–0.99.

In this section, comparisons are made between different PDC calculations over specific values of interest, because this is a common way of reporting group adherence. Results are described for ACE inhibitors, unless otherwise specified, with any differences noted in the other medication classes.

### PDC differences

The proportion of people prescribed ACE inhibitors whose PDC was at least 0.8 ranges, depending on follow-up period, from 53 to 70% under PDC1 assumptions, 84–90% under PDC2 assumptions and 88–100% under PDC3 assumptions, with similar ranges in statins and thyroid hormones. Thus, the assumption that people have had their prescription discontinued after their last refill (the difference between PDC2 and PDC1) makes a substantial difference to the percentage of people with high adherence. The further assumption that a priori defined large gaps between refills are not due to sub-optimal implementation (PDC3) brings the percentage close to 100% in some circumstances.

There is a similar pattern in the proportion of people prescribed ACE inhibitors whose PDC is at least 0.9, but the percentages are smaller: 43–63% (PDC1), 67–79% (PDC2), and 71–91% (PDC3). Again, these ranges are similar in statins and thyroid hormones.

### PDC3 gap sensitivity

PDC3 is sensitive to the definition of a large gap between medication refills. The proportion of people on ACE inhibitors with PDC3 ≥ 0.8 ranges from 88 to 90% under conservative gap exclusions, 92–95% under moderate exclusions, and 97–100% under liberal exclusions, with similar ranges in statins and thyroid hormones.

### Effect of follow-up lengths

Longer follow-up lengths tended to, but did not always, lead to lower proportions of people with high PDC1 and PDC2 values. The proportion with PDC1 ≥ 0.8 over a 3- 6- 12- and any-month period was 70, 61, 53 and 54%, and the proportion with PDC2 ≥ 0.8 was 90, 85, 84 and 85%.

In contrast, longer follow-up tended to lead to higher proportions of people with high PDC3 values, especially with liberal and moderate gap definitions. The proportion with PDC3 (0.5) ≥ 0.8 over 3-, 6-, 12- and any-month periods was 97, 99, 99 and 100%, and PDC3 (1.0) ≥ 0.8 was 92, 92, 94 and 95%. The trend was unclear under conservative definitions: PDC (1.5) ≥ 0.8 was 90, 88, 89 and 90. There were similar percentages and trends in the other two drug classes.

### Comparison between drug classes

**Fig. 4:**
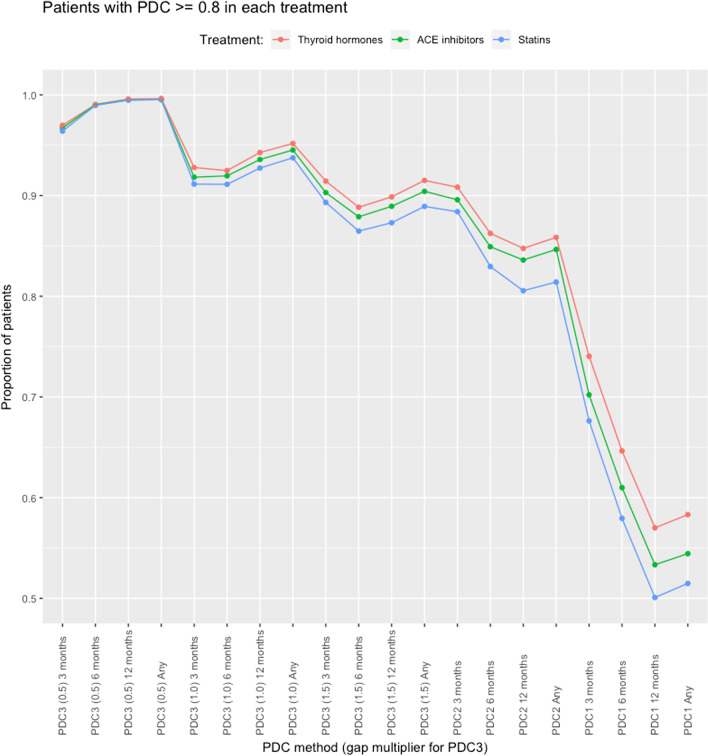
Comparison of proportion of patients with PDC ≥ 0.8 in each drug class (thyroid hormones, ACE inhibitors, and statins)

The three drug classes follow similar patterns of adherence across all PDC definitions. Adherence to thyroid hormones was higher than to ACE inhibitors and statins: all PDCs ≥ 0.8 and PDCs ≥ 0.9 were higher (see Fig. 4), which may reflect the differing demographics (younger and more female).

### Comparison between patient characteristics

Additional file 1: Fig. S1a–c shows the proportion of patients PDC ≥ 0.8 in different subgroups of patients. The patterns are similar across the different definitions of PDCs (lines are parallel), although for those definitions with more coverage the differences between subgroups tend to be smaller. The larger differences seem to be between age groups and ordering platforms. The patterns for the other treatments are similar.

## Discussion

The aim of this paper was to propose and evaluate adaptations of the PDC algorithm when only data on orders placed and dispensed from a single pharmacy available. The alternative PDC algorithms address different questions and incorporate different assumptions about people’s prescription needs and potential for meeting them with other pharmacy providers. The algorithms are designed to help in real-world scenarios where an organisation, such as a payor or an online pharmacy provider, may want to estimate the PDC as a quality indicator of medication adherence, a supplier’s performance and/or to identify the potential for improvement in patient services.

When applied to the online pharmacy dataset, there were differences in estimations of adherence depending on the algorithm applied. As expected, PDC1 was lower than either PDC2 or PDC3, and PDC2 was lower than PDC3. Differences in adherence rates will be dependent on the data themselves—in very adherent group of people, estimates of adherence will be similar across the algorithms, but when more people are sub-optimally adherent, the differences between PDC 1, 2 and 3 will be greater.

Limited prior evidence exists on alternative approaches to traditional PDC calculations to increase the accuracy of adherence estimates in real-world settings [20]. This study proposed and evaluated alternative PDC algorithms which have wider applicability to settings where a particular supplier may have incomplete data. These include online pharmacy providers that are unable to access and link people’s redeemed prescriptions with the original prescribing data. Alternative approaches to calculate PDC have practical value because of a growing availability and use of online pharmacies and increased individual choice. Assessing the quality of these services, and eventually comparing services from different providers, is vital and requires a flexible yet proven tool kit, including adapted measures to evaluate medication adherence.

An example of how these alternative PDCs can help online pharmacy providers comes from NHS England, where people who opt-in to electronic prescription services can select a ‘nominated pharmacy’ to which their electronic prescriptions are sent by default [19]. ‘Nomination switching’ away from a previously selected pharmacy can occur without the incumbent nominated pharmacy being informed. People also have the right to request a paper-based prescription for any reason at any time, even if they have opted-in to electronic prescription services [19]. These patient-level choices can thus create gaps in pharmacy data records that are not attributable to medication nonadherence.

PDC is already recognised as a quality indicator by a growing number of organisations, particularly in the United States (US). In addition to its endorsement by the PQA [17] PDC metrics are required by the Centers for Medicare & Medicaid Services (CMS) for renin–angiotensin system antagonists, statins, and all diabetes drugs [22]. Utilising these alternative PDCs present an opportunity for better understanding of medication adherence behaviours and usage in the online pharmacy setting and will highlight areas where improvements may be needed to support people with their prescribed medication, i.e. reminder services and prompts to engage with the patient about any potential issues with their medication.

Pharmacies could use the data generated from the different PDC calculations in several different ways. In every situation where there is a possibility that a gap in supply stems from nonadherence (e.g. when using PDC1, 2 or 3), there is an opportunity for intervention. A gap in supply should therefore always trigger contact with the patient. While reminders may help when nonadherence is due to a lack of capability (such as forgetting to collect the refill, for example because of being busy, or cognitive impairment), a reminder alone will be less effective when nonadherence stems from people’s concerns about potential adverse effects or doubts about the need for treatment [23]. Routinely measuring barriers to treatment, for example through online questionnaires, would enable pharmacies to provide adherence interventions tailored to the specific needs of individual patients. A randomised controlled trial showed that an intervention delivered by pharmacists over the telephone could significantly increase adherence, reduce medicine-related problems, and increase people’s perceptions of their personal need for medicine relative to their concerns about adverse effects [24]. This type of intervention could be adapted by online pharmacies for delivery via digital channels.

This study has shown that PDCs could also be used as quality indicators of the suppliers’ performance in covering customer demand. From the supplier perspective, PDC1 and PDC2 most likely measure performance in covering total customer demand, while PDC3 measures performance in covering actively engaged customer demand.

The current algorithm is designed to calculate the PDC of one drug only, or one drug class, at any one time. However, if an individual were taking more than one drug, or drug class, for a specific condition, it would be helpful to be able to calculate a combined PDC. A simple approach would be to create a combined PDC from the average (simple or weighted) across the PDC calculated separately for each drug. However, other definitions of combined coverage could be more insightful, i.e. proportion of days where the individual has supplies of all their drugs or proportion of days where they have supplies of at least one of the drugs. Future adaptation and testing of the algorithm would be needed to evaluate this.

If used as a proxy estimate of medication adherence, these PDCs must be interpreted with care. PDC1 and PDC2 assume the worst-case scenario that people are not taking their medication when there is no evidence of supply from the supplier in question. On the contrary, PDC3 assumes that people continue their coverage with other providers or are taking a medically justified treatment interruption. None of these assumptions can be verified using provider data alone, but PDC1 or PDC2 could be used as lower bounds for adherence and a version of PDC3 for a reasonable upper bound. Since PDC3 has a parameter that is not fixed (the magnitude of gaps to be censored), we always suggest conducting sensitivity analysis when reporting results.

Having alternative PDC algorithms may also help researchers to define adherence in accordance with the ABC taxonomy [6]. The traditional PDC1 calculation considers that a large gap in medication supply is due to nonadherence, however it does not provide information about the specific adherence behaviour in question, e.g. whether nonadherence is due to sub-optimal implementation (missing doses, or taking less medicine than prescribed), or discontinuation despite the medical need for treatment [6]. In contrast, because PDC2 and PDC3 do not consider periods beyond last refill by the supplier, use of either one of these algorithms is unlikely to conflate discontinuation with sub-optimal implementation. Use of PDC2 and PDC3 may therefore be a more appropriate choice when implementation is the behaviour of interest. Moreover, when it can be reasonably assumed that large gaps are due to the person obtaining medication from an alternative supplier, PDC3 could enhance the measurement of implementation.

Limitations of this study include the absence of a second source of data (such as patient interviews, another measure of adherence (e.g. self-report) or clinical data (e.g. blood pressure readings or blood test results) all of which could provide information about the validity of the different PDC measurements. We found that PDC was sensitive to the definition of a large gap between supplies, therefore it is important to establish reasonable assumptions for different prescriptions. Further studies using interview methods are required to investigate what happens in large gaps. Following people over a period of interest, for example the first year of treatment, and comparing PDC measurements with a second measure of adherence, such as self-report, would also be useful to examine patterns of adherence (e.g. initiation, implementation, and persistence) in relation to PDC measurements.

## Conclusions

The availability of alternative PDC algorithms allows suppliers and researchers to select the approach most relevant to their research question and the necessary assumptions. The PDCs outlined in this paper could enable organisations with dispensing data to track the progress of their patients over time and to evaluate the effect of interventions targeted at improving medication adherence. Increased use of algorithms to analyse real-world data might help improve our understanding of adherence in real-world settings, highlighting the true challenges and opportunities for intervention. Improving medication adherence has the potential to both improve health outcomes for individuals, as well as reduce medicines wastage and healthcare costs.

## Supplementary Information

**Additional file 1: Figure S1. **Comparison of PDCs by patient characteristics.

**Additional file 2: Table S1.** Three PDC algorithms applied to a real-world dataset of people taking ACE inhibitors, statins and thyroid hormones.

## Data Availability

The dataset analysed during the current study is not publicly available because it comes from a privately owned, commercial database and is restricted by a privacy policy. The dataset can be requested under a confidentiality agreement. Our specifications in the manuscript provide sufficient guidance for readers to develop the adapted Proportion of Days Covered (PDC) algorithms. Our code is not currently packaged as an R package, however we would be happy to share our code on request.

## Abbreviations

ACE: Angiotensin-converting enzyme
CMS: Centers for Medicare and Medicaid Services
MPR: Medication possession ratio
NHS: National Health Service
PDC: Proportion of days covered
POI: Period of interest
PQA: Pharmacy Quality Alliance
UK: United Kingdom
US: United States
WHO: World Health Organization
