# Supplementary material for: Estimating proportion of days covered (PDC) using real-world online medicine suppliers’ datasets
Source: J Pharm Policy Pract. 2021 Dec 29;14:113. doi: 10.1186/s40545-021-00385-w (PMC8715592; doi:10.1186/s40545-021-00385-w)

## **Figure S1:** Comparison of PDCs by patient characteristics

1. Patients with PDC >=0.8 in ACE inhibitors and follow up of 12 months


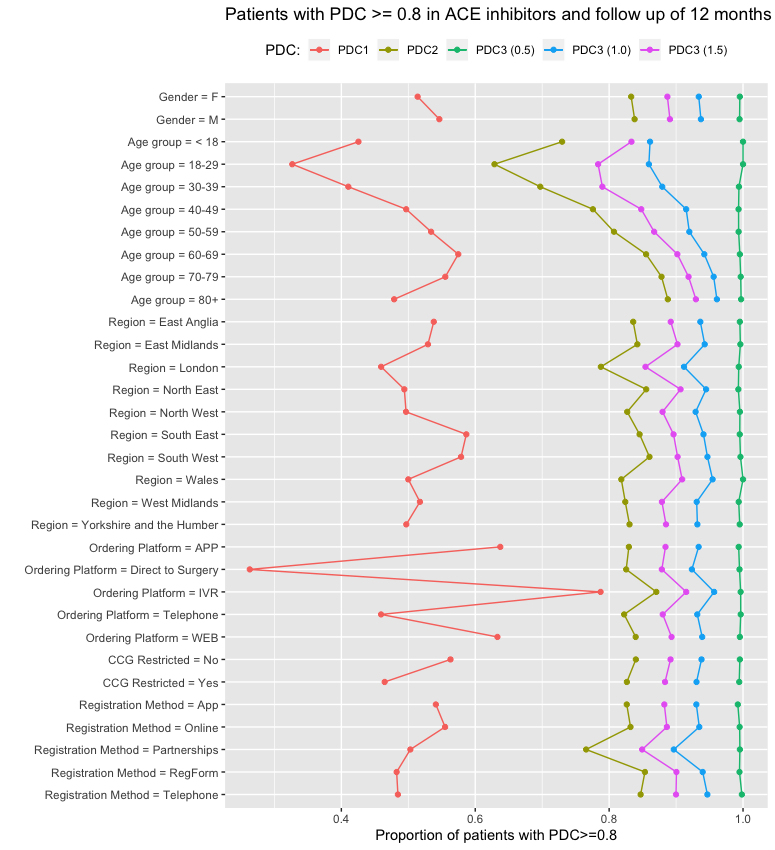


1. Patients with PDC >=0.8 in Statins and follow up of 12 months


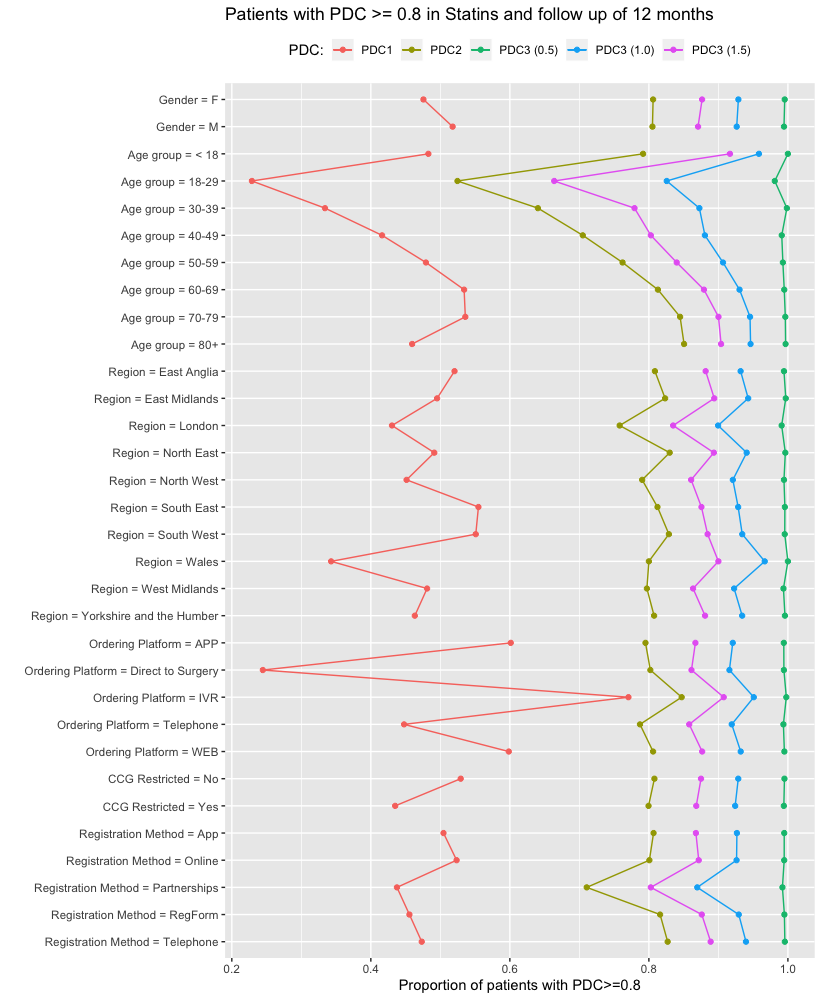


1. Patients with PDC >=0.8 on thyroid hormones and follow up of 12 months


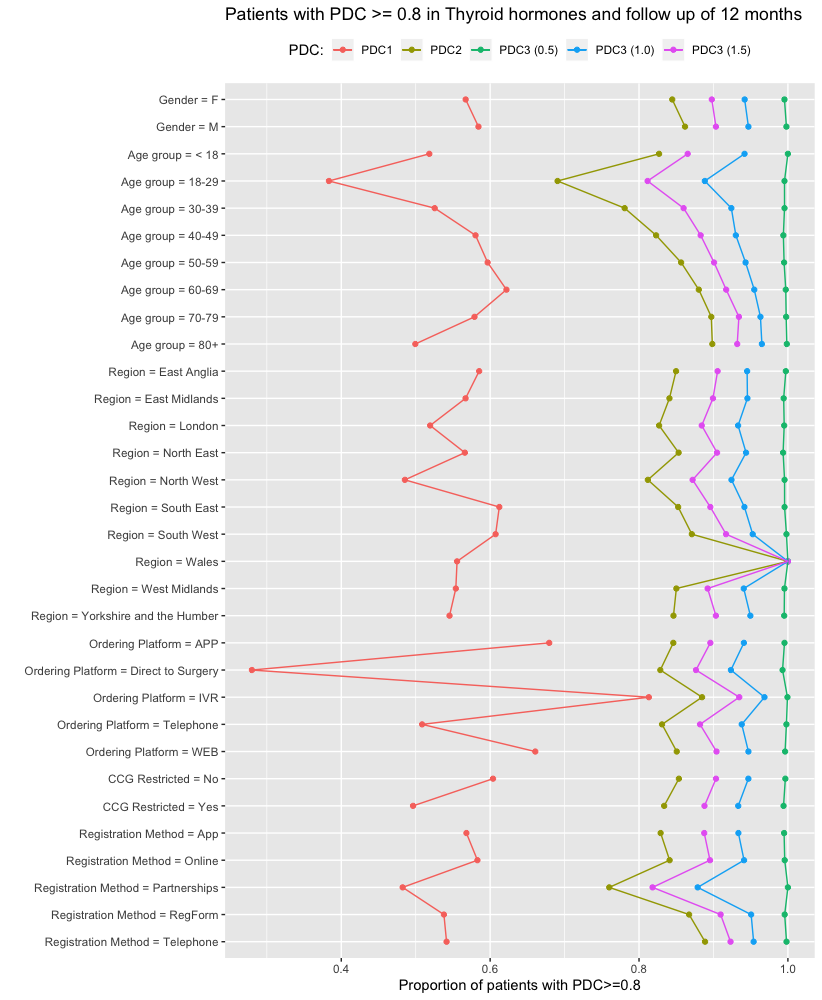

Supplement: Supplementary file 1 — Additional file 1: Figure S1. Comparison of PDCs by patient characteristics. [file 40545_2021_385_MOESM1_ESM.docx]
