# Supplementary material for: Estimating proportion of days covered (PDC) using real-world online medicine suppliers’ datasets
Source: J Pharm Policy Pract. 2021 Dec 29;14:113. doi: 10.1186/s40545-021-00385-w (PMC8715592; doi:10.1186/s40545-021-00385-w)
Supplement: Supplementary file 2 — Additional file 2: Table S1. Three PDC algorithms applied to a real-world dataset of people taking ACE inhibitors, statins and thyroid hormones. [file 40545_2021_385_MOESM2_ESM.docx]

**Table S1:** Three PDC algorithms applied to a real-world dataset of people taking ACE inhibitors, statins and thyroid hormones

| **Medicine** | **type** | **Follow** | **N** | **Mean** | **SD** | **Q.05** | **Q.10** | **Q.50** | **P>=0.8** | **P>=0.9** |
| --- | --- | --- | --- | --- | --- | --- | --- | --- | --- | --- |
| ACE inhibitors | PDC1 | 3 months | 61317 | 0.84 | 0.21 | 0.31 | 0.62 | 0.93 | 70% | 63% |
| ACE inhibitors | PDC1 | 6 months | 56875 | 0.76 | 0.28 | 0.15 | 0.31 | 0.91 | 61% | 51% |
| ACE inhibitors | PDC1 | 12 months | 44420 | 0.68 | 0.33 | 0.08 | 0.15 | 0.84 | 53% | 43% |
| ACE inhibitors | PDC1 | Any | 65786 | 0.67 | 0.35 | 0.08 | 0.11 | 0.85 | 54% | 44% |
| ACE inhibitors | PDC2 | 3 months | 48373 | 0.94 | 0.10 | 0.70 | 0.79 | 1.00 | 90% | 79% |
| ACE inhibitors | PDC2 | 6 months | 48087 | 0.91 | 0.12 | 0.64 | 0.73 | 0.96 | 85% | 71% |
| ACE inhibitors | PDC2 | 12 months | 38083 | 0.90 | 0.13 | 0.62 | 0.73 | 0.95 | 84% | 67% |
| ACE inhibitors | PDC2 | Any | 54184 | 0.91 | 0.13 | 0.62 | 0.73 | 0.96 | 85% | 69% |
| ACE inhibitors | PDC3 (0.5) | 3 months | 46503 | 0.96 | 0.06 | 0.82 | 0.87 | 1.00 | 97% | 87% |
| ACE inhibitors | PDC3 (0.5) | 6 months | 46016 | 0.96 | 0.05 | 0.85 | 0.89 | 0.98 | 99% | 87% |
| ACE inhibitors | PDC3 (0.5) | 12 months | 37055 | 0.96 | 0.05 | 0.87 | 0.90 | 0.98 | 99% | 89% |
| ACE inhibitors | PDC3 (0.5) | Any | 52789 | 0.97 | 0.04 | 0.87 | 0.90 | 0.98 | 100% | 91% |
| ACE inhibitors | PDC3 (1.0) | 3 months | 47522 | 0.95 | 0.08 | 0.75 | 0.82 | 1.00 | 92% | 81% |
| ACE inhibitors | PDC3 (1.0) | 6 months | 46934 | 0.94 | 0.08 | 0.76 | 0.82 | 0.97 | 92% | 77% |
| ACE inhibitors | PDC3 (1.0) | 12 months | 37569 | 0.94 | 0.07 | 0.78 | 0.83 | 0.96 | 94% | 77% |
| ACE inhibitors | PDC3 (1.0) | Any | 53493 | 0.94 | 0.07 | 0.79 | 0.84 | 0.97 | 95% | 79% |
| ACE inhibitors | PDC3 (1.5) | 3 months | 48025 | 0.94 | 0.09 | 0.73 | 0.80 | 1.00 | 90% | 80% |
| ACE inhibitors | PDC3 (1.5) | 6 months | 47544 | 0.93 | 0.10 | 0.70 | 0.78 | 0.96 | 88% | 73% |
| ACE inhibitors | PDC3 (1.5) | 12 months | 37785 | 0.92 | 0.09 | 0.73 | 0.79 | 0.96 | 89% | 71% |
| ACE inhibitors | PDC3 (1.5) | Any | 53790 | 0.93 | 0.09 | 0.74 | 0.80 | 0.97 | 90% | 74% |
| Statins | PDC1 | 3 months | 93217 | 0.83 | 0.22 | 0.31 | 0.59 | 0.92 | 68% | 60% |
| Statins | PDC1 | 6 months | 86834 | 0.74 | 0.29 | 0.15 | 0.31 | 0.89 | 58% | 48% |
| Statins | PDC1 | 12 months | 68119 | 0.66 | 0.33 | 0.08 | 0.15 | 0.80 | 50% | 40% |
| Statins | PDC1 | Any | 100164 | 0.66 | 0.35 | 0.07 | 0.10 | 0.82 | 51% | 41% |
| Statins | PDC2 | 3 months | 71607 | 0.93 | 0.11 | 0.69 | 0.78 | 0.99 | 88% | 78% |
| Statins | PDC2 | 6 months | 72213 | 0.90 | 0.13 | 0.62 | 0.71 | 0.96 | 83% | 68% |
| Statins | PDC2 | 12 months | 57709 | 0.89 | 0.14 | 0.58 | 0.69 | 0.94 | 81% | 63% |
| Statins | PDC2 | Any | 81398 | 0.89 | 0.15 | 0.57 | 0.69 | 0.95 | 81% | 65% |
| Statins | PDC3 (0.5) | 3 months | 68433 | 0.96 | 0.06 | 0.82 | 0.87 | 1.00 | 96% | 86% |
| Statins | PDC3 (0.5) | 6 months | 68389 | 0.96 | 0.05 | 0.85 | 0.89 | 0.98 | 99% | 87% |
| Statins | PDC3 (0.5) | 12 months | 55637 | 0.96 | 0.05 | 0.86 | 0.89 | 0.97 | 99% | 89% |
| Statins | PDC3 (0.5) | Any | 78704 | 0.96 | 0.04 | 0.87 | 0.90 | 0.98 | 100% | 90% |
| Statins | PDC3 (1.0) | 3 months | 70032 | 0.95 | 0.09 | 0.75 | 0.81 | 1.00 | 91% | 80% |
| Statins | PDC3 (1.0) | 6 months | 70062 | 0.93 | 0.08 | 0.75 | 0.81 | 0.97 | 91% | 75% |
| Statins | PDC3 (1.0) | 12 months | 56574 | 0.93 | 0.08 | 0.78 | 0.82 | 0.96 | 93% | 74% |
| Statins | PDC3 (1.0) | Any | 79960 | 0.94 | 0.07 | 0.78 | 0.83 | 0.97 | 94% | 77% |
| Statins | PDC3 (1.5) | 3 months | 70918 | 0.94 | 0.10 | 0.70 | 0.79 | 0.99 | 89% | 79% |
| Statins | PDC3 (1.5) | 6 months | 71142 | 0.92 | 0.10 | 0.69 | 0.77 | 0.96 | 86% | 71% |
| Statins | PDC3 (1.5) | 12 months | 57005 | 0.92 | 0.09 | 0.72 | 0.78 | 0.95 | 87% | 68% |
| Statins | PDC3 (1.5) | Any | 80520 | 0.92 | 0.09 | 0.73 | 0.79 | 0.96 | 89% | 70% |
| Thyroid hormones | PDC1 | 3 months | 28608 | 0.86 | 0.20 | 0.31 | 0.62 | 0.96 | 74% | 68% |
| Thyroid hormones | PDC1 | 6 months | 26678 | 0.78 | 0.27 | 0.15 | 0.31 | 0.92 | 65% | 56% |
| Thyroid hormones | PDC1 | 12 months | 20869 | 0.71 | 0.32 | 0.08 | 0.15 | 0.87 | 57% | 47% |
| Thyroid hormones | PDC1 | Any | 30568 | 0.70 | 0.34 | 0.08 | 0.12 | 0.89 | 58% | 48% |
| Thyroid hormones | PDC2 | 3 months | 22108 | 0.95 | 0.10 | 0.71 | 0.81 | 1.00 | 91% | 82% |
| Thyroid hormones | PDC2 | 6 months | 22693 | 0.92 | 0.12 | 0.66 | 0.75 | 0.97 | 86% | 74% |
| Thyroid hormones | PDC2 | 12 months | 17982 | 0.91 | 0.13 | 0.64 | 0.74 | 0.96 | 85% | 70% |
| Thyroid hormones | PDC2 | Any | 25312 | 0.91 | 0.13 | 0.64 | 0.74 | 0.97 | 86% | 71% |
| Thyroid hormones | PDC3 (0.5) | 3 months | 21371 | 0.97 | 0.06 | 0.84 | 0.88 | 1.00 | 97% | 88% |
| Thyroid hormones | PDC3 (0.5) | 6 months | 21752 | 0.97 | 0.05 | 0.86 | 0.89 | 0.99 | 99% | 89% |
| Thyroid hormones | PDC3 (0.5) | 12 months | 17530 | 0.97 | 0.04 | 0.87 | 0.90 | 0.98 | 100% | 91% |
| Thyroid hormones | PDC3 (0.5) | Any | 24730 | 0.97 | 0.04 | 0.88 | 0.91 | 0.99 | 100% | 92% |
| Thyroid hormones | PDC3 (1.0) | 3 months | 21783 | 0.95 | 0.08 | 0.76 | 0.84 | 1.00 | 93% | 84% |
| Thyroid hormones | PDC3 (1.0) | 6 months | 22211 | 0.94 | 0.08 | 0.77 | 0.83 | 0.98 | 92% | 79% |
| Thyroid hormones | PDC3 (1.0) | 12 months | 17754 | 0.94 | 0.07 | 0.79 | 0.84 | 0.97 | 94% | 79% |
| Thyroid hormones | PDC3 (1.0) | Any | 25018 | 0.95 | 0.07 | 0.80 | 0.85 | 0.98 | 95% | 81% |
| Thyroid hormones | PDC3 (1.5) | 3 months | 21974 | 0.95 | 0.09 | 0.74 | 0.82 | 1.00 | 91% | 82% |
| Thyroid hormones | PDC3 (1.5) | 6 months | 22472 | 0.93 | 0.10 | 0.70 | 0.79 | 0.97 | 89% | 76% |
| Thyroid hormones | PDC3 (1.5) | 12 months | 17851 | 0.93 | 0.09 | 0.74 | 0.80 | 0.97 | 90% | 74% |
| Thyroid hormones | PDC3 (1.5) | Any | 25149 | 0.94 | 0.08 | 0.75 | 0.81 | 0.97 | 92% | 76% |

NOTE:

Q.05 = Percentile 5: 95% of patients have a PDC greater than this

Q.10 = Percentile 10: 90% of patients have a PDC greater than this

Q.50 = Percentile 10: 50% of patients have a PDC greater than this

P>0.8 = % of patients have a PDC greater than 0.8

P>0.9 = % of patients have a PDC greater than 0.9
